# An Engineered Herpesvirus Activates Dendritic Cells and Induces Protective Immunity

**DOI:** 10.1038/srep41461

**Published:** 2017-02-02

**Authors:** Yijie Ma, Min Chen, Huali Jin, Bellur S. Prabhakar, Tibor Valyi-Nagy, Bin He

**Affiliations:** 1 Department of Microbiology and Immunology, University of Illinois College of Medicine, Chicago, Illinois, USA; 2 Institute of Viral Hepatitis, The Second Affiliated Hospital, Chongqing Medical University, Chongqing 400010, China; 3 Department of Pathology, University of Illinois College of Medicine, Chicago, Illinois, USA

## Abstract

Herpes simplex viruses (HSV) are human pathogens that switch between lytic and latent infection. While attenuated HSV is explored for vaccine, the underlying event remains poorly defined. Here we report that recombinant HSV-1 with a mutation in the γ_1_34.5 protein, a virulence factor, stimulates dendritic cell (DC) maturation which is dependent on TANK-binding kinase 1 (TBK1). When exposed to CD11^+^ DCs, the mutant virus that lacks the amino terminus of γ_1_34.5 undergoes temporal replication without production of infectious virus. Mechanistically, this leads to sequential phosphorylation of interferon regulatory factor 3 (IRF3) and p65/RelA. In correlation, DCs up-regulate the expression of co-stimulatory molecules and cytokines. However, selective inhibition of TBK1 precludes phosphorylation of IRF3 and subsequent DC activation by the γ_1_34.5 mutant. Herein, the γ_1_34.5 mutant is immune-stimulatory and non-destructive to DCs. Remarkably, upon immunization the γ_1_34.5 mutant induces protection against lethal challenge by the wild type virus, indicative of its vaccine potential. Furthermore, CD11^+^ DCs primed by the γ_1_34.5 mutant *in vivo* mediate protection upon adoptive transfer. These results suggest that activation of TBK1 by engineered HSV is crucial for DC maturation, which may contribute to protective immunity.

Herpes simplex viruses (HSV) are human pathogens responsible for a spectrum of diseases, including genital herpes, encephalitis, and keratitis that often leads to blindness^1^,^2^,^3^. HSV typically infects the mucosal tissues where it undergoes viral entry, gene expression, DNA replication, assembly and egress. In this process, the virus sequentially expresses an array of viral proteins that work coordinately, resulting in a productive cycle. Following lytic infection, HSV invades the peripheral nervous system and subsequently establishes latency in the ganglia^1^,^2^,^3^. Viral reactivation occurs intermittently, which is the lifelong source of recurrent lesions^2^,^3^. Currently, no vaccine is available to treat HSV infections.

Several lines of evidence suggest that dendritic cells (DCs) restrict HSV infection^4^,^5^,^6^,^7^. As sentinels, DCs recognize HSV through distinct pathways^8^,^9^. For example, Toll-like receptor (TLR) 9 detects the viral genome to induce production of interferon-α. TLR3 binds viral RNA and recruits an adaptor TIR domain-containing adapter inducing IFN-β (TRIF), which triggers TANK-binding kinase 1(TBK1) and I-κB kinase, activating interferon regulatory factor 3 (IRF3) and NF-κB, respectively^5^,^10^. Alternative mechanisms also exist in DCs to detect HSV. These include cyclic GAMP synthase and DDX41 that sense intracellular viral DNA and induce cytokine expression^11^,^12^. However, HSV replication can cripple DCs and subsequent T cell activation. It has been suggested that HSV-1 interaction with immature DCs results in down-regulation of costimulatory molecules and cytokines^13^,^14^,^15^. In addition, HSV-1 perturbs the function of mature DCs by degradation of a cell surface molecule CD83^16^,^17^. Moreover, HSV reduces the chemokine receptors CCR7 and CXCR4 and impairs DC migration to the respective chemokine ligands CCL19 and CXCL12^18^. It appears that interplay of HSV and DCs represents a regulatory interface in HSV infection^14^.

The γ_1_34.5 gene, located in the inverted repeats of the HSV genome, encodes a virulence factor^19^. In HSV-infected cells onset of viral DNA replication activates dsRNA-dependent protein kinase, which shuts off viral protein synthesis^20^. As a countermeasure, the γ_1_34.5 protein redirects protein phosphatase 1 to dephosphorylate eIF-2α^21^. Accordingly, the γ_1_34.5 null mutant is avirulent^22^. Based on this property, derivatives of γ_1_34.5 mutants are developed as viral vaccines^23^,^24^,^25^ or oncolytic agents^26^,^27^,^28^,^29^,^30^,^31^,^32^. Nonetheless, the functional features associated with the γ_1_34.5 mutants remain obscure. We recently noted that the γ_1_34.5 protein negatively modulates TBK1. As such, a γ_1_34.5 mutant with deletion of the amino-terminus fails to prelude cytokine expression and is attenuated^33^. Here, we report that the γ_1_34.5 mutant without the amino-terminus activates DCs through TBK1. Furthermore, immunization with the γ_1_34.5 mutant protects against lethal challenge from wild type virus, which relies on DCs.

## Results

### An HSV-1 mutant that lacks the amino-terminus of γ_1_34.5 infects DCs

**Figure 1:**
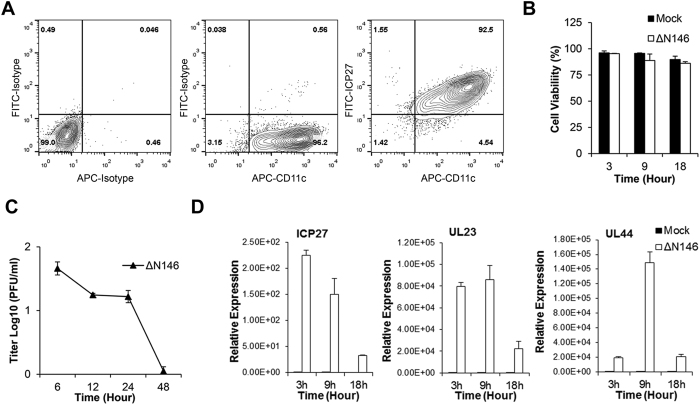
(**A**) Viral infection of immature DCs. Mouse bone marrow derived DCs cultured in the presence of GM-CSF were infected with ΔN146 virus (5 PFU/cell) for 3 h. Infectivity was determined by examining ICP27 expression in CD11c^+^ cells via flow cytometry as described in Methods. (**B**) Effects of viral infection on cell viability. Immature DCs were mock infected or infected with the ΔN146 virus (5 PFU/cell). Cell viability was measured by the trypan blue exclusion method at the indicated time points. (**C**) Viral growth in immature DCs. Cells were infected with ΔN146 mutant (0.05 PFU/cell). At different time points, cells were harvested and freeze-thawed three times. Virus titers were determined on Vero cells via plaque assay. (**D**) Viral gene expression in immature DCs. Cells were mock infected or infected with the ΔN146 virus. Total RNAs were extracted and subjected to qRT-PCR to evaluate ICP27 (α gene), UL23 (β gene), and UL44 (γ gene) mRNA levels. The data were normalized to 18 S rRNA, and fold induction was calculated as described in Methods. Results are expressed as relative expression with standard deviations among triplicate samples.

To characterize an HSV-1 mutant that lacks the amino-terminal domain of γ_1_34.5, we evaluated virus infection of DCs. As such, immature murine DCs, generated from bone marrow in the presence of GM-CSF, were exposed to ΔN146 for 3 h. The expression of ICP27, an immediate-early (α) gene of HSV-1, was examined by fluorescence-activated cell sorter analysis (FACS) to measure the infectivity. As shown in Fig. 1A, 92.5% of cells were double positive for both CD11c and ICP27 at 3 h post infection, indicative of an efficient infection. The cell viability assay indicated that over 85% of DCs infected with ΔN146 remained viable throughout the course of 18 h infection (Fig. 1B). Notably, the viral yield reduced to undetectable level at 48 h post infection, suggesting an impaired production of infectious viruses (Fig. 1C).

As HSV gene expression proceeds in a sequential manner^1^, we analyzed ICP27 (α gene), UL23 (β gene) and UL44 (γ gene) in ΔN146. Total RNA from infected DCs was subjected to quantitative real-time PCR analysis. As illustrated in Fig. 1D, in the course of infection, the expression of ICP27 peaked at 3 h post infection and gradually decreased as infection progressed to the late phase. In contrast, expression of the β gene UL23 which encodes the thymidine kinase remained at a similar level initially but reduced to a lower level at 18 h post infection. The γ gene UL44 which encodes glycoprotein C was expressed at a lower level during early stage of the infection (3 h). Its expression peaked at 9 h and then decreased to a minimum level at 18 h post infection. Thus, although impaired for viral production the amino terminal deletion mutant of γ_1_34.5 retains the capacity to express immediate early, early and late genes.

### The γ_1_34.5 truncation mutant stimulates dendritic cell maturation

**Figure 2:**
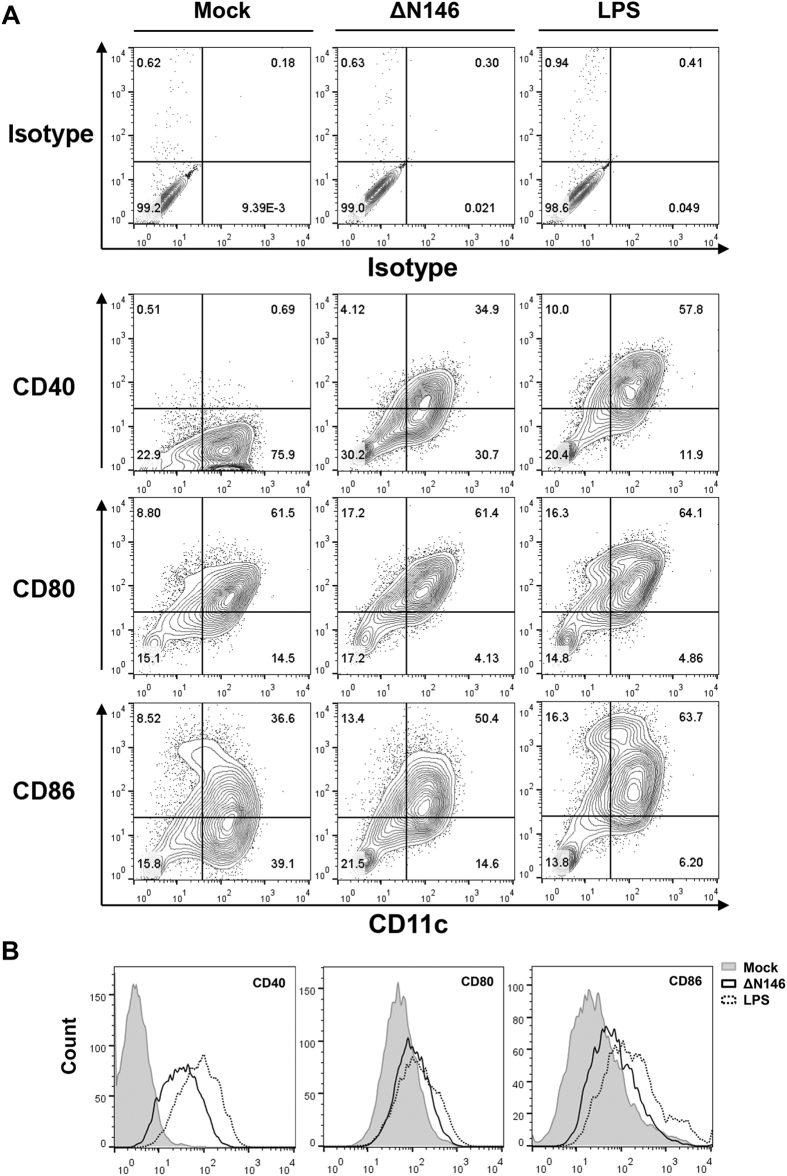
The ΔN146 mutant activates DCs *ex vivo*. (**A**) Immature DCs were mocked infected, infected with ΔN146 (5 PFU/cell) or treated with LPS (500 ng/ml). At 12 hours post infection, cells were stained with CD11c and assayed for CD40, CD80 or CD86 positivity by flow cytometry. (**B**) Immature BMDCs were infected or treated as in (**A**) and assayed by flow cytometry. Cells were gated on CD11c positive population and assayed for CD40, CD80 or CD86 fluorescence intensity.

To assess the impact of the γ_1_34.5 mutant on DC activation, we determined the expression of cell surface molecules. Immature DCs were either mock infected, infected with ΔN146 (5 PFU/cell) or treated with lipopolysaccharide (LPS, 500 ng/ml) for 12 h, and subsequently subjected to FACS analysis for the expression of CD40, CD80 and CD86. As expected, LPS treatment augmented CD40-postive and CD86-positive CD11c^+^ DCs as compared to the mock infection (Fig. 2A). Similarly, ΔN146 infection resulted in significant increase in the number of CD40-positive and CD86-positive CD11c^+^ DCs compared to the mock group. Consistent with this, both ΔN146 infection and LPS treatment dramatically stimulated the expression of CD40 and CD86 as indicated by enhanced fluorescence intensities (Fig. 2B). Although the proportion of CD80-positive CD11c^+^ DCs was similar in all treatment groups (Fig. 2A), ΔN146 infection and LPS stimulated higher surface expression of CD80 molecules than mock infection (Fig. 2B).

**Figure 3:**
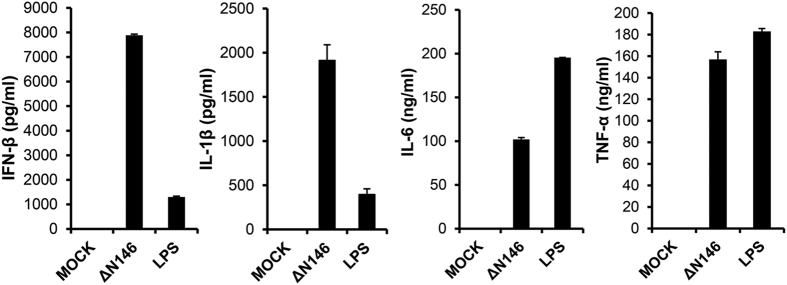
ΔN146 infection stimulates cytokine expression in immature DCs. Immature DCs were mocked infected, infected with ΔN146 (5 PFU/cell) or treated with LPS (500 ng/ml). At 12 h post infection, Cell supernatants were collected and assayed by ELISA for the expression of cytokines IFN-β, IL-1β, IL-6 and TNF-α. The results are representative of at least three independent experiments with standard deviations among triplicate samples.

We next analyzed the cytokine production in the supernatants from the DCs by ELISA. As shown in Fig. 3, ΔN146-infected cells, similar to the LPS-treated cells, expressed and secreted copious levels of type I interferon β (IFN-β), IL-1β, IL-6 and tumor necrosis factor α (TNF-α) 12 h post infection whereas these cytokines were barely detectable in supernatants from mock infected cells. The different magnitudes of cytokine levels between ΔN146-infected and LPS-treated cells are possibly due to the fact that LPS specifically activates Toll-like receptor 4 (TLR4) signaling whereas HSV infection triggers a complex program involving multiple innate immune signaling pathways^34^,^35^. These results suggest that the γ_1_34.5 truncation mutant stimulates DC maturation.

### The γ_1_34.5 mutant triggers activation of IRF3 and NF-κB in immature DCs

**Figure 4:**
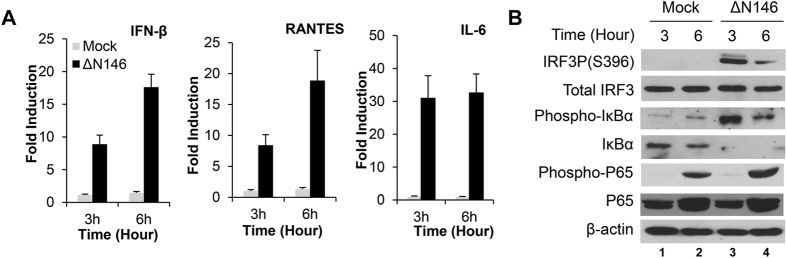
The ΔN146 mutant induces innate antiviral signaling in immature DCs. (**A**) DCs were either mock infected or infected with ΔN146 at 5 PFU/cell. At indicated time points, total RNA extracted from cells was subjected to quantitative real-time PCR amplification for expression of IFN-β, RANTES, or IL-6. The data were normalized to 18 S rRNA and expressed as relative expression with standard deviations among triplicate samples. (**B**) DCs were infected as described in panel A. Cell lysates were subjected to immunoblotting analysis with antibodies against IRF3, phosphorylated IRF3 (Ser396), IκBα, phosphorylated IκBα, P65, and phosphorylated P65, respectively, at 3 h and 6 h post infection.

To assess innate immune signaling in DC response to ΔN146, we examined cytokine expression in immature DCs during the early stage of infection. Figure 4A shows that ΔN146 infection triggered a robust increase in mRNA levels of IFN-β, RANTES and IL-6 at 3 h post infection. Such response became more evident at 6 h post infection. In parallel experiments, we evaluated the impact of ΔN146 on IRF3 and NF-κB in immature DCs. As shown in Fig. 4B, ΔN146 infection drastically induced the phosphorylation of IRF3 at serine 396, a hallmark of IRF3 activation. In stark contrast, phosphorylated IRF3 was undetectable in mock infected cells, although the total level of IRF3 proteins remained comparable in all groups. Furthermore, ΔN146 induced increased phosphorylation and degradation of Iκ-Bα as compared to mock infected cells, indicating that the canonical NF-κB pathway was also activated in immature DCs early in infection. We conclude that ΔN146 rapidly activates IRF3 and NF-κB signaling pathways, which contributes to the maturation of DCs.

### Inhibition of TBK1 precludes DC maturation in response to virus infection

**Figure 5:**
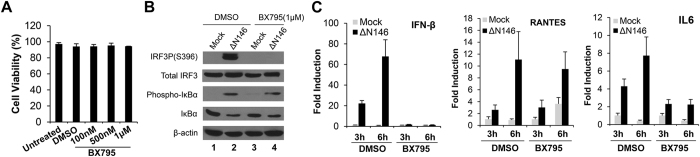
TBK1 is required for ΔN146 induced signaling in immature DCs. (**A**) Effect of TBK1 inhibitor on cell viability. Immature DCs were left untreated or treated with DMSO or TBK1 inhibitor BX795 at indicated concentrations. At 14 h after treatment, cell viability was measured by the trypan blue exclusion method. The results are mean values of duplicate samples with standard deviations. (**B**) Effect of TBK1 inhibitor on the activation of innate immune signaling pathways triggered by ΔN146 mutant. Immature DCs were treated and infected as B. At 3 hours post infection, cells were collected and subjected to immunoblotting using antibodies against IRF3, phosphorylated IRF3 (Ser396), IκBα, phosphorylated IκBα, and β-actin respectively. (**C**) Effect of TBK1 inhibitor on the expression of antiviral genes and inflammatory cytokines. Immature DCs were left untreated or treated with DMSO or TBK1 inhibitor BX795 (1 μM) for 1 hour. Subsequently cells were either mock infected or infected by ΔN146 mutant (5 PFU/cell) in the presence or absence of BX795 (1 μM). At 3 and 6 h post infection, RNAs were isolated from the cells and assayed by quantitative real-time PCR for the expression of IFN-β, RANTES and IL6. The data were normalized to the level of 18 S rRNA and expressed as relative expression with standard deviations among triplicate samples.

To define the mechanism of ΔN146 action, we focused on TBK1. As such, we carried out a series of experiments by using BX795 that potently inhibits TBK1 activity^36^. Cell toxicity assay showed that this compound had no effect on DC viability when treatment dose was increased up to 1 μM (Fig. 5A). Under this assay condition, BX795 completely blocked the phosphorylation of IRF3 in DCs upon exposure to ΔN146 (Fig. 5B). On the other hand, BX795 had no impact on phosphorylation and degradation of Iκ-Bα, suggesting a selective inhibition of TBK1. Further analysis showed that BX795 effectively inhibited the induction of IFN-β by ΔN146 as measured by qRT-PCR (Fig. 5C). BX795 also reduced the induction of IL-6 expression, suggesting a pivotal role of TBK1. However, BX795 did not block RANTES induction, indicating that ΔN146 triggers its expression via a factor other than TBK1.

**Figure 6:**
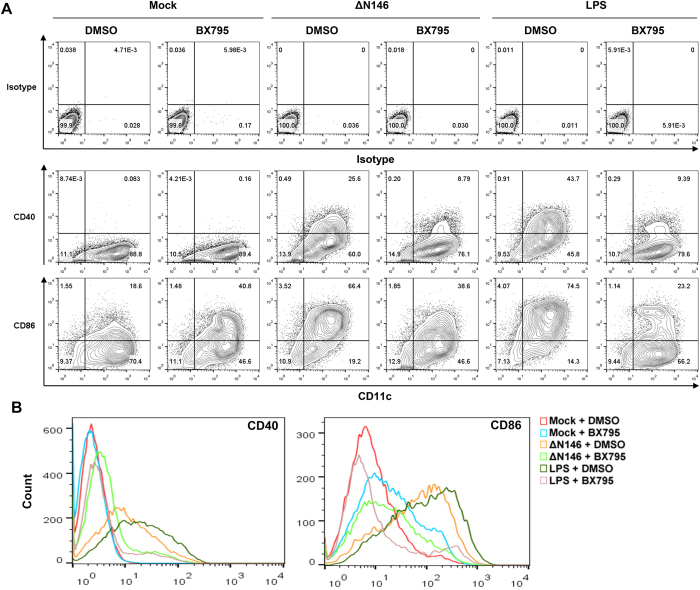
Inhibition of TBK1 impairs DC activation induced by ΔN146 *ex vivo*. (**A**) Immature DCs were left untreated or treated with DMSO or TBK1 inhibitor BX795 (1 μM) for 1 h. Cells were then mock infected, infected by ΔN146 mutant (5 PFU/cell) or stimulated with LPS (500 ng/ml) in the presence or absence of BX795 (1 μM). At 12 h post-infection, cells were stained with CD11c and assayed for CD40 or CD86 positivity by flow cytometry. (**B**) DCs were treated as panel A and assayed by flow cytometry. Cells were gated on CD11c positive population and assayed for CD40 or CD86 fluorescence intensity.

**Figure 7:**
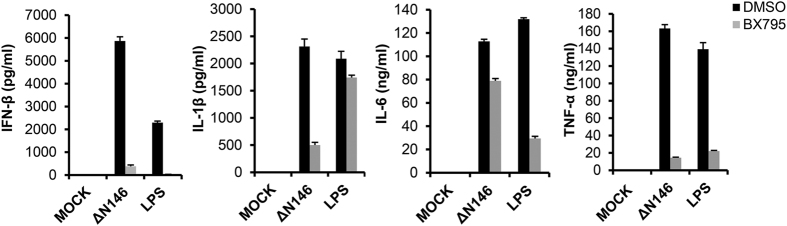
Inhibition of TBK1 signaling severely impairs cytokine production in ΔN146 infected DCs. Immature DCs were mock infected, infected with ΔN146 (5 PFU/cell) or stimulated with LPS (500 ng/ml) in the presence of DMSO or BX795 for 12 h. Cell supernatants were assayed for IFN-β, IL-1β, IL-6, and TNF-α by ELISA. The results are from triplicate samples with standard deviations.

We examined the effect of TBK1 signaling on the expression of cell surface molecules in DCs upon infection with ΔN146. Immature DCs were mock infected, infected with ΔN146 in the presence or absence of BX795. LPS was included as a control. As shown in Fig. 6A, CD40 was barely detectable in mock infected CD11c^+^ DCs. ΔN146 infection and LPS stimulation significantly increased the population of CD11c^+^ CD40^+^ cells to 25.6% and 43.7%, respectively. Addition of BX795, however, remarkably reduced the percentage of CD11c^+^ CD40^+^ cells to 8.79% and 9.39% in ΔN146 infected and LPS stimulated cells, respectively. A similar trend was also observed for CD86^+^ DCs although a higher basal expression was seen mock infected cells. In accordance with this, ΔN146 and LPS robustly augmented the surface expression of CD40 and CD86 in mock treated cells, while in the presence of BX795 DCs stimulated by ΔN146 and LPS exhibited a marginal or no increase in the surface expression of CD40 and CD86 compared to the mock group (Fig. 6B). In addition, BX795 inhibited the production of IFN-β and TNF-α in DCs induced by ΔN146 and LPS (Fig. 7). Interestingly, the TBK1 inhibitor strongly suppressed ΔN146-induced IL-1β production and LPS-induced IL-6 production while modestly impairing ΔN146-induced IL-6 expression and LPS-induced IL-1β expression. These results suggest that TBK1 signaling mediates DC maturation in response to ΔN146 infection.

### The γ_1_34.5 mutant induces protective immunity via DCs *in vivo*

**Figure 8:**
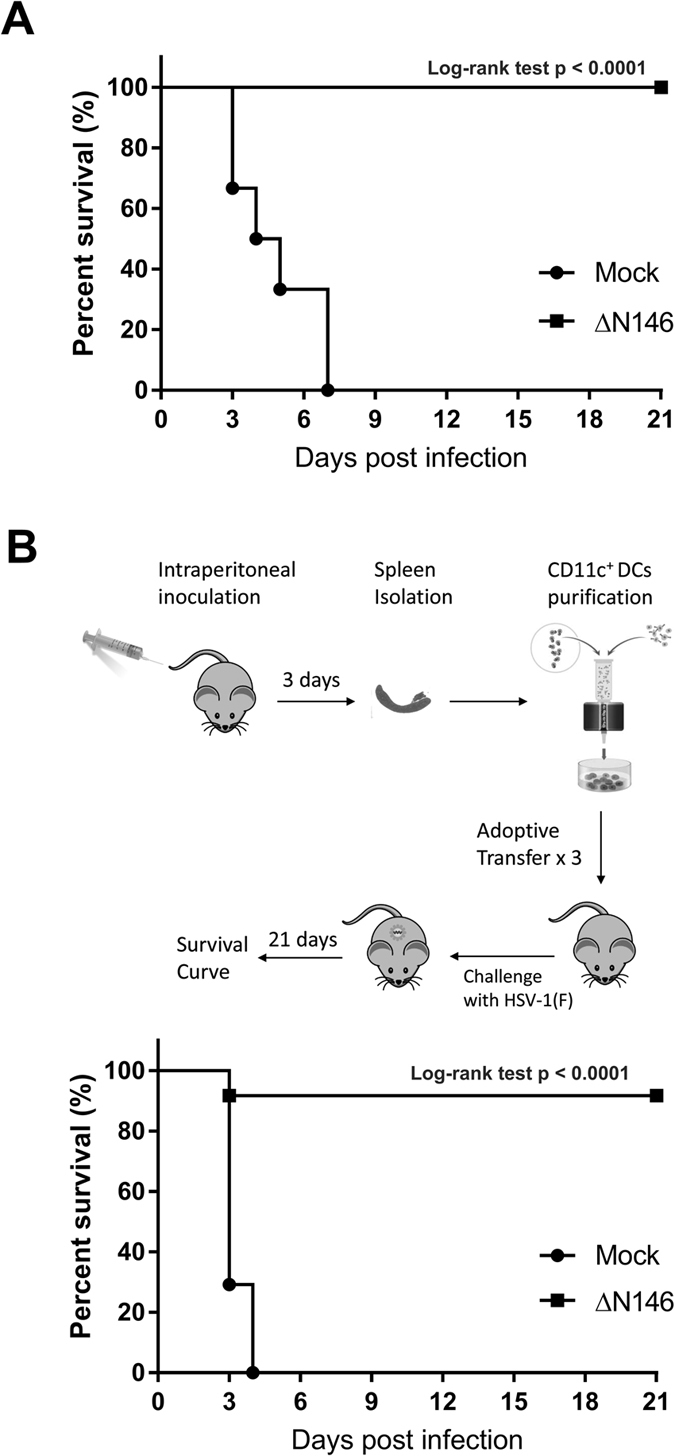
DCs mediate protective immunity elicited by ΔN146 vaccination against wild type HSV-1. (**A**) Mice were mock inoculated or inoculated with ΔN146 at the dose of 1 × 10^5 ^PFU intraperitoneally. Two weeks after immunization, the mice were challenged with HSV-1(F) (1 × 10^7 ^PFU) intranasally and monitored over a 21 day period. The survival rates were analyzed by Kaplan-Meier plots (n = 24, log-rank test p < 0.0001) using GraphPad Prism 7. (**B**) Mice were mock inoculated or inoculated with ΔN146 (1 × 10^5 ^pfu) intraperitoneally with a repeat on day 14. Three days after, CD11c^+^ DCs, isolated from spleen of immunized mice, were transferred into naïve mice three times every other day. Next day after the last transfer, mice were challenged with HSV-1(F) (1 × 10^7 ^pfu) and monitored for additional 21 days. The survival rates were analyzed by Kaplan-Meier plots (n = 24, log-rank test p < 0.0001) using GraphPad Prism 7.

Based on above results, we asked whether ΔN146 has a vaccine potential. Accordingly, we inoculated BALB/c mice with mock or ΔN146 (1 × 10^5 ^PFU) intraperitoneally. Two weeks after inoculation, these mice were intranasally challenged with a lethal dose of HSV-1(F) (1 × 10^7 ^PFU) and monitored for 21 days. As shown in Fig. 8A, mice inoculated with ΔN146 exhibited a 100% protection against lethal HSV-1 challenge whereas mock group started dying after day 2 and no mouse survived beyond day 7. As ΔN146 stimulates DC maturation *ex vivo*, we hypothesized that DCs may play a critical role in mediating ΔN146-induced protective immunity. To test this, mice were inoculated with mock or ΔN146 intraperitoneally, with a repeated inoculation on day 14. Three days after, CD11c^+^ DCs were isolated from spleens and subsequently transferred into naïve mice three times every other day. Mice were challenged with HSV-1(F) one day later and monitored for an additional period of 21 days. As shown in Fig. 8B, all mice in the mock group died within 4 days of lethal challenge. In stark contrast, 90% of recipient mice with DCs derived from the ΔN146 immunized group survived throughout over a period of 21 days. These data suggest that upon immunization ΔN146 induces protective immunity against lethal HSV infection through dendritic cells.

## Discussion

In this study, we show that an HSV-1 mutant that harbors an N-terminal truncation in the γ_1_34.5 protein is able to infect DCs and induce phosphorylation of IRF3 and RelA/p65. This is accompanied by upregulation of IFN-α/β, inflammatory cytokines, and co-stimulatory molecules, a hallmark of DC activation. Intriguingly, suppression of TBK1 function by a chemical inhibitor dramatically impaired DC maturation. Furthermore, immunization with this mutant protects against wild type infection through DCs. These results suggest a model that an engineered γ_1_34.5 mutant can induce protective immunity via TBK1.

Our work suggests that deletion of the amino-terminus from γ_1_34.5 renders the virus immune-stimulatory in DCs. Although unable to produce infectious virus the γ_1_34.5 amino-terminal deletion mutant infected DCs efficiently. This is suggested by the fact that over 90% DCs were susceptible to infection, with little reduction in cell viability. Notably, as infection progressed the virus expressed ICP27 (α gene), UL23 (β gene), and UL44 (γ gene), indicating a temporal viral replication. In correlation, the DCs secreted elevated levels of IFN-β, IL-1β, IL-6 and TNF-α. Similarly, DCs expressed higher levels of maturation markers CD40 and CD86. It seems that deletion of the amino-terminus from γ_1_34.5 rendered the virus immune-stimulatory. Indeed, the γ_1_34.5 mutant sequentially induced phosphorylation of IRF3 and RelA/p65 at 3 h post infection of DCs. While it suggests an early event, the viral component(s) involved is to be defined. Accumulating evidence suggests that cyclic GAMP synthase (cGAS) is critically important in recognition of HSV-1^11^. Additionally, DDX41 detects HSV-1 in DCs^12^. We suspect that the γ_1_34.5 mutant may trigger cGAS or DDX41 that activates IRF3 and NF-κB. Alternatively, viral RNA intermediates produced upon infection may stimulate the RIG-I or TLR3 pathway in DCs^35^. These models are not necessarily mutually exclusive. Work is in progress to explore these possibilities.

TBK1 sits at the center of innate immune pathways that usually induce type I IFN responses^35^. We noted that the γ_1_34.5 mutant activates DCs, which relies on TBK1 activity. Two lines of evidence support this argument. First, chemical inhibition of TBK1 blocked phosphorylation of IRF3 but not I-κB degradation induced by the γ_1_34.5 mutant, suggesting a selective inhibition of TBK1. Second, it sharply reduced the expression of CD40 and CD86 upon exposure to the γ_1_34.5 mutant. This was mirrored by a reduction in the expression of IFN-β, IL-1β, IL-6 and TNF-α. In this respect, it is surprising that inhibition of TBK1 reduced the expression of inflammatory cytokines. A simple explanation is that TBK1 dominantly controls inflammatory cytokine expression in DCs infected with the γ_1_34.5 mutant. Interpreted in this framework, it is notable that a cross talk exists where TBK1 phosphorylates NF-κB that drives inflammatory cytokine expression^37^. Herein, such mechanism may operate in DCs in response to HSV infection. These studies underscore the importance of TBK1 in DCs activation upon exposure to the γ_1_34.5 mutant.

It is noteworthy the γ_1_34.5 mutant induces protective immunity *in vivo*. With single immunization, the γ_1_34.5 mutant conferred complete protection against lethal challenge over a period of three weeks. While additional work is required, it suggests a vaccine potential of the γ_1_34.5 mutant. Relevant to this are observations that the γ_1_34.5 mutant devoid of the amino-terminal domain is attenuated and stimulates DC maturation. In this context, we observed that DCs from mice immunized with the γ_1_34.5 mutant confer protection upon adoptive transfer. As DCs play a role in limiting HSV-1 infection^4^,^5^,^6^,^7^, these results lend support to the model that the γ_1_34.5 mutant primes DCs, which translates into protective immunity. At this stage, the precise way by which the γ_1_34.5 mutant induces protection is unknown. An attractive possibility is that upon immunization the γ_1_34.5 amino-terminal deletion mutant may directly engage with DCs *in vivo*. In doing so, it likely activates TBK1, a component that is required for induction of antigen-specific B and T cells^38^. Our future work will focus on the precise mechanism by which the engineered γ_1_34.5 mutant to confer protection *in vivo*.

## Methods

### Mice

BALB/c mice were purchased from Harlan Sprague-Dawley Inc. and housed under specific-pathogen-free conditions in biosafety level 2 containment. Groups of 5-week-old mice were selected for this study. Mice protocols were approved by the institutional office of animal care and biosafety committee. Experiments were performed in accordance with the guidelines of the University of Illinois at Chicago.

### Cells and viruses

Myeloid CD11c+ DCs were generated as previously described^15^. Briefly, bone marrow cells were isolated from the tibia and femur bones of BALB/c mice. Following red blood cell lysis and washing, progenitor cells were plated in DC complete medium which is RPMI 1640 medium (Invitrogen, Auckland, New Zealand) supplemented with 10% fetal bovine serum (FBS), 0.1 mM nonessential amino acids, 1 mM sodium pyruvate, and 20 ng/ml granulocyte-macrophage colony-stimulating factor (GM-CSF; BioSource, Camarillo, CA). Cells were supplemented with fresh medium every other day. For adoptive transfer and viral replication experiments, DCs were first positively selected for surface CD11c expression using magnetic beads (Miltenyi Biotech, Auburn, CA) to give a ≥97% pure population of CD11c+ major histocompatibility complex class II-positive (MHC-II+) cells. In recombinant virus ΔN146, the region encoding amino acids 1 to 146 of γ_1_34.5 is deleted^33^.

### Viral infection and DC transfer

DCs were infected with ΔN146 at indicated MOI in RPMI 1640 supplemented with 1% fetal bovine serum (FBS), 0.1 mM nonessential amino acids, and 1 mM sodium pyruvate. At different time points after infection, cells were harvested for analysis. For lethal challenge experiment, mice were first anesthetized and mock inoculated or inoculated intraperitoneally with 1 × 10^5 ^PFU of ΔN146. Two weeks after virus inoculation, mice were intranasally challenged with 1 × 10^7 ^PFU of wild-type HSV-1(F). Mice were monitored daily for overall health and sacrificed when symptoms of encephalitis appeared. For *in vivo* transfer analysis, mice were mock inoculated or inoculated with ΔN146 (1 × 10^5 ^pfu) intraperitoneally, with a repeated inoculation on day 14. Single splenocyte suspensions were prepared three days after. And CD11c+ DCs were isolated and purified by using the CD11c magnetic beads according to the manufacturer’s protocol (Miltenyi Biotech). The cells, with a purity of 96–98%, were transferred into naïve mice (5 × 10^6 ^cells/mouse) three times intraperitoneally on day 1, 3, and 5, respectively. On day 6 after the first transfer the mice were challenged with HSV-1(F) and monitored for 3 weeks

### Plaque assay

To determine the titer of infectious virus, virus-infected DCs were harvested and freeze-thawed three times. Samples were serially diluted in 199 v medium, and viral yields were titrated on Vero cells at 37 °C^33^.

### Immunoblot analysis and ELISA

To analyze protein expression, cells were harvested and solubilized in disruption buffer containing 50 mM Tris-HCl (pH 7.0), 5% 2-mercaptoethanol, 2% SDS, and 2.75% sucrose. Samples were then subjected to electrophoresis on denaturing polyacrylamide gels, transferred to nitrocellulose membranes, blocked with 5% nonfat milk, and reacted with antibodies against β-actin (Sigma), IRF3 (Santa Cruz Biotechnology), phosphorylated IRF3 (pSer396) (Cell Signaling Technology, Inc.), IκBα and phosphorylated IκBα (Santa Cruz Biotechnology), p65/RelA and phosphorylated p65/RelA (pSer536) (Cell Signaling Technology, Inc.). The membranes were rinsed in phosphate-buffered saline and reacted with donkey anti-rabbit immunoglobulin conjugated to horseradish peroxidase. Protein bands were detected by enhanced chemiluminescence (Bio-Rad). To perform enzyme-linked immunosorbent assays (ELISA), supernatants of cell culture were collected and analyzed using mouse tumor necrosis factor alpha (TNF-α) and IFN-β ELISA kits (R&D systems), and mouse interleukin-6 (IL-6) and IL-1β ELISA kits (eBioscience) according to the manufacturer’s instructions.

### Flow cytometry

DCs were harvested at the time of assay and washed once with FACS buffer (D-PBS containing 0.5% BSA and 2 mM EDTA), followed by blocking non-specific binding with anti-mouse CD16/CD32 (eBioscience, San Diego, CA). To examine cell surface molecules, cells were stained with isotype-matched antibodies, anti-CD11c-PE or anti-CD11c-APC, anti-CD40-FITC, anti-CD80-FITC and anti-CD86-FITC antibodies (eBioscience, San Diego, CA) for 30 min on ice. For intracellular ICP27 staining, cells were fixed in 4% paraformaldehyde (Sigma) and permeabilized in permeabilizing buffer (eBioscience, San Diego, CA). Cells were blocked with 5% normal goat serum (Sigma), incubated with anti-HSV-1 ICP27 (Virusys, Sykesville, MD), and stained with goat anti-mouse FITC-conjugated antibody (Santa Cruz Biotech, CA). Samples were processed and screened using a FACSCalibur fluorescence-activated cell sorter (FACS) and data were analyzed using FlowJo VX software.

### Quantitative real-time PCR assay

Quantitative real-time PCR assay was performed as previously described^33^. Total RNA was harvested from cells using an RNeasy kit (Qiagen) and subjected to DNase I digestion (New England BioLabs). Quantitative real-time PCR was performed using an Applied Biosystems ABI Prism 7900HT instrument with ABI Fast SYBR green Master Mix (Applied Biosystems), and data were normalized to endogenous control 18 S rRNA. Relative expression or fold induction was calculated using 2^−ΔΔCt^ method with the normalized Ct value of the untreated or mock treated sample at the earliest time point being the baseline. Primers for mouse genes were chosen according to the recommendation of the qPrimerDepot database^39^. Primer sequences were as follows: mouse IFN-β, AATTTCTCCAGCACTGGGTG and AGTTGAGGACATCTCCCACG; mouse RANTES, CTGCTGCTTTGCCTACCTCT and CACTTCTTCTCTGGGTTGGC; mouse IL-6,; 18 s rRNA, CCTGCGGCTTAATTTGACTC and AACCAGACAAATCGCTCCAC; HSV-1 ICP27, CCTTTCTCCAGTGCTACCTG and GCCAGAATGACAAACACGAAG; HSV-1 UL23, AGAAAATGCCCACGCTACTG and CACCTGCCAGTAAGTCATCG; HSV-1 UL44, CGACTACAGCGAGTACATCTG and CGATTCCAATCCCCACCC.

## Additional Information

**How to cite this article**: Ma, Y. *et al*. An Engineered Herpesvirus Activates Dendritic Cells and Induces Protective Immunity. *Sci. Rep.* **7**, 41461; doi: 10.1038/srep41461 (2017).

**Publisher's note:** Springer Nature remains neutral with regard to jurisdictional claims in published maps and institutional affiliations.
